# M4Raw: A multi-contrast, multi-repetition, multi-channel MRI k-space dataset for low-field MRI research

**DOI:** 10.1038/s41597-023-02181-4

**Published:** 2023-05-10

**Authors:** Mengye Lyu, Lifeng Mei, Shoujin Huang, Sixing Liu, Yi Li, Kexin Yang, Yilong Liu, Yu Dong, Linzheng Dong, Ed X. Wu

**Affiliations:** 1 College of Health Science and Environmental Engineering, Shenzhen Technology University, Shenzhen, China; 2 Guangdong-Hongkong-Macau Institute of CNS Regeneration, Key Laboratory of CNS Regeneration (Ministry of Education), Jinan University, Guangzhou, China; 3 Department of Neurosurgery, Shenzhen Samii Medical Center, Shenzhen, China; 4 Laboratory of Biomedical Imaging and Signal Processing, The University of Hong Kong, Hong Kong, China; 5 Department of Electrical and Electronic Engineering, The University of Hong Kong, Hong Kong, China

**Keywords:** Brain, Biomedical engineering

## Abstract

Recently, low-field magnetic resonance imaging (MRI) has gained renewed interest to promote MRI accessibility and affordability worldwide. The presented M4Raw dataset aims to facilitate methodology development and reproducible research in this field. The dataset comprises multi-channel brain k-space data collected from 183 healthy volunteers using a 0.3 Tesla whole-body MRI system, and includes T1-weighted, T2-weighted, and fluid attenuated inversion recovery (FLAIR) images with in-plane resolution of ~1.2 mm and through-plane resolution of 5 mm. Importantly, each contrast contains multiple repetitions, which can be used individually or to form multi-repetition averaged images. After excluding motion-corrupted data, the partitioned training and validation subsets contain 1024 and 240 volumes, respectively. To demonstrate the potential utility of this dataset, we trained deep learning models for image denoising and parallel imaging tasks and compared their performance with traditional reconstruction methods. This M4Raw dataset will be valuable for the development of advanced data-driven methods specifically for low-field MRI. It can also serve as a benchmark dataset for general MRI reconstruction algorithms.

## Background & Summary

Magnetic resonance imaging (MRI) is a powerful medical imaging technology for clinical diagnosis of various diseases. However, MRI accessibility is low and highly uneven around the world, with the majority of MRI scanners concentrated in high-income countries, leaving approximately 70% of the world’s population with little or no access to MRI^1–6^. Low-field MRI under 1 Telsa (T) has gained renewed interest^6–14^ as a potential solution to this problem due to its significantly lower cost for purchase, installation, and maintenance compared to high-field MRI systems. In addition to the economic considerations, low-field MRI has a number of intrinsic advantages compared to high-field MRI, including improved patient comfort, low sensitivity to metallic implants, fewer image susceptibility artifacts, and extremely low radiofrequency specific absorption rate (SAR)^1–6^. However, despite improvements in MRI hardware since the 1980s, the key limiting factor at low field remains the signal-to-noise ratio (SNR) per unit time. This often results in long scan times and compromised image quality, hindering the adoption of low-field MRI in areas that require fast imaging and high SNR.

Recently, data-driven methods, particularly deep learning-based approaches, have emerged as a potential solution to the SNR problem at low field. In the field of computer vision, data-driven methods have rapidly evolved to outperform traditional methods by a wide margin in many low-level tasks, such as denoising, deblurring, and super-resolution^15–17^. These methods have been deployed in digital cameras and mobile phones for daily use with robust performance. Similar trends have been seen in the field of MRI reconstruction^18–20^. For example, variational neural network (VarNet) based methods^21^ have been proposed and evaluated in a number of MRI applications^21–24^, showing superior performance in accelerating scans and boosting SNR.

Training data are critical for the development of data-driven methods. In comparison to natural images, MRI data are rare, and most public MRI datasets^25–28^ only include magnitude images, which lack important phase and multi-channel information necessary for realistic MRI reconstruction tasks^29^. Furthermore, the few existing multi-channel k-space datasets^30–33^ were all acquired using high-field MRI systems, which have different signal and noise characteristics from low-field systems. The lack of publicly available low-field MRI data has become a barrier for researchers to enter this field or reproduce different approaches of previous studies.

To address this gap, we present a new multi-channel k-space dataset acquired using low-field MRI. It contains brain data from 183 subjects, each with 18 axial slices and 3 contrasts: T1-weighted (T1w), T2-weighted (T2w), and fluid attenuated inversion recovery (FLAIR). Importantly, each contrast includes two or three repetitions, resulting in more than 1,000 volumes in total that can be used in various ways by the MRI community. We name this multi-contrast, multi-repetition, multi-channel MRI raw k-space dataset as M4Raw for low-field MRI research. In this paper, we describe the method for producing this dataset and demonstrate its potential uses in denoising and parallel imaging reconstruction.

## Methods

**Fig. 1:**
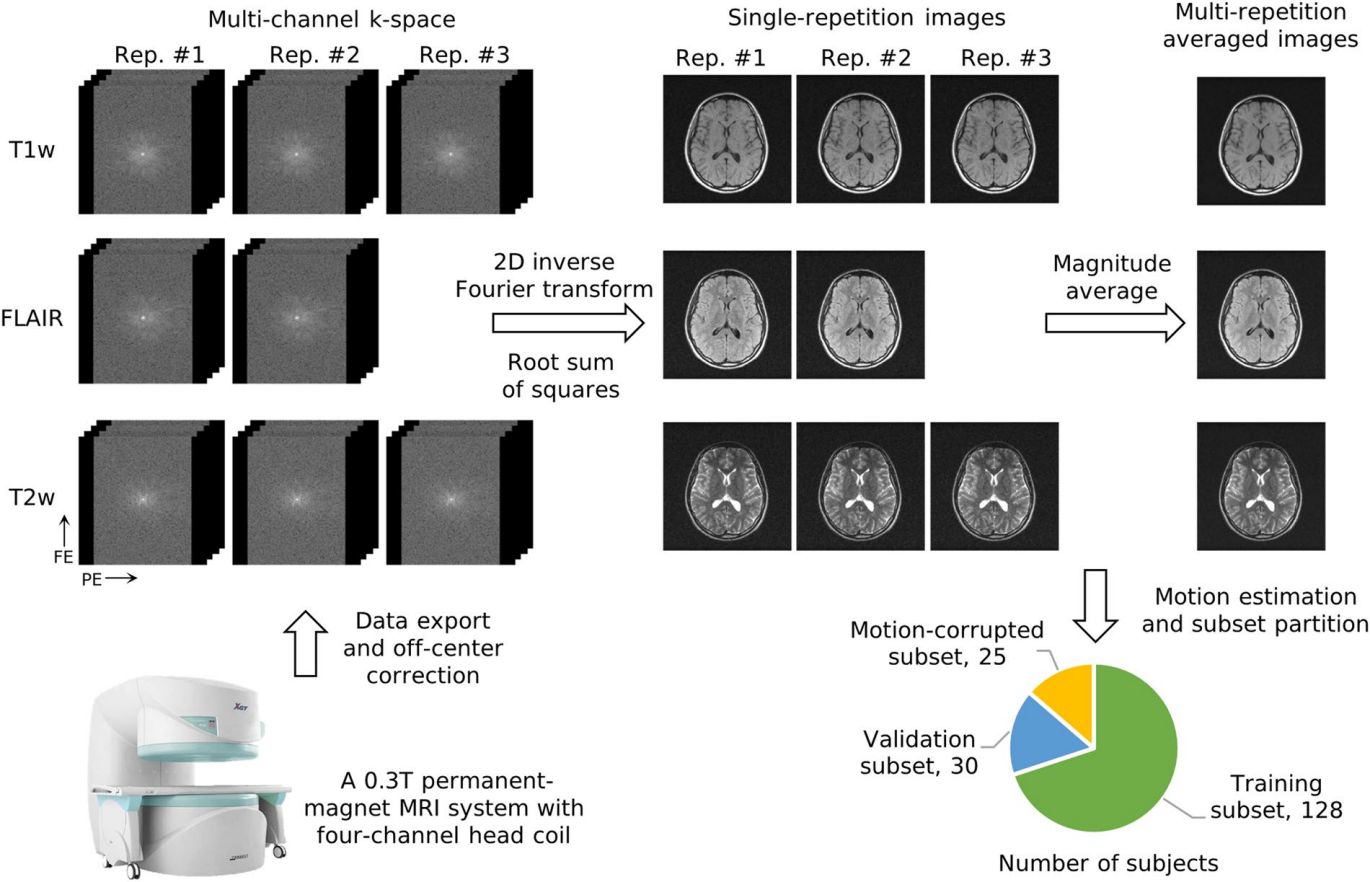
General workflow to produce the M4Raw dataset. Multi-contrast, multi-repetition, multi-slice, multi-channel k-space data were acquired from 183 healthy volunteers using a 0.3 T MRI system equipped with a four-channel head coil. The final dataset includes a training subset of 128 subjects, a validation subset of 30 subjects, and a motion-corrupted subset of 25 subjects. Phase encoding (PE) and frequency encoding (FE) directions are marked.

The general workflow to produce the M4Raw dataset is illustrated in Fig. 1. Multi-contrast, multi-repetition, multi-channel MRI k-space data were collected from 183 healthy volunteers using a 0.3 T MRI system with a four-channel head coil. Single-repetition images were generated by applying the inverse Fourier transform to the k-space data and combining the coil signals using the root sum of squares. Multi-repetition averaged images were then obtained by calculating the magnitude average of individual repetitions for each contrast. The final dataset comprises a training subset of 128 subjects, a validation subset of 30 subjects, and a motion-corrupted subset of 25 subjects.

### Imaging protocol

A total of 183 healthy volunteers were enrolled in the study with written informed consent, following approval by the Institutional Review Board of Shenzhen Technology University (reference number: SZTULL012021005). All participants were cognizant of the nature of the study, and provided consent for their materials to be made publicly available in anonymized form as part of the written consent process. The majority of the participants were college students (aged 18 to 32, mean = 20.1, standard deviation (std) = 1.5; 116 males, 67 females). Axial brain MRI data were obtained from each subject using a clinical 0.3 T scanner (Oper-0.3, Ningbo Xingaoyi) equipped with a four-channel head coil. This scanner is a classical open type permanent magnet-based whole-body system. Three common sequences were used: T1w, T2w, and FLAIR, each acquiring 18 slices with a thickness of 5 mm and an in-plane resolution of 0.94 × 1.23 mm^2^. To facilitate flexible research applications, T1w and T2w data were acquired with three individual repetitions and FLAIR with two repetitions. The decision to only acquire two repetitions for FLAIR was made due to its long scan time per repetition. Acquiring more repetitions would result in more than 6.5 min total scan time and increase vulnerability to motion artifacts. T1w scans were performed first, followed by FLAIR scans, and lastly T2w scans, all with identical multi-slice geometry planning. The complete imaging parameters are summarized in Table 1.

**Table 1:** MRI sequence parameters for data acquisition.

	Sequence type	Matrix size	TR/TE	Scan time per repetition	No. of repetitions	Echo train length	Echo spacing	Common parameters
T1w	Spin echo	256 × 195	500/18.4 ms	1 min 38 s	3	1	—	Field-of-view: 240 × 240 mm^2^ No. of slices: 18 Slice thickness/gap: 5/1 mm Sampling bandwidth: 31.25 KHz Phase encoding in left-right direction
T2w	Fast spin echo	256 × 195	5500/128 ms	1 min 12 s	3	15	16.0 ms	
FLAIR	Inversion recovery fast spin echo	256 × 198	7500/98 ms; TI = 1655 ms	2 min 15 s	2	11	16.3 ms	

### Data processing

The k-space data from individual repetitions were exported from the scanner console without averaging. The corresponding raw images were in scanner coordinate space and may be off-centered due to patient positioning. To correct this, an off-center distance was estimated along the left-right direction for each subject using the vendor DICOM images, and the k-space data were multiplied by a corresponding linear phase modulation. The k-space matrices were then converted to Hierarchical Data Format Version 5 (H5) format^34^, with imaging parameters stored in the H5 file header in an ISMRMRD-compatible format^35^. The items in the DICOM headers were selectively transferred to the H5 headers, ensuring subject anonymization in accordance with the DICOM Basic Application Level Confidentiality Profile. We applied Retain Device Identity, UIDs, Longitudinal Temporal Information, and Institution Identity Options while removing all items related to subject identity, such as subject name, personal ID, and date of birth. The k-space dimensions were arranged in the same manner as the fastMRI dataset^30^, allowing existing codes for fastMRI to be run on M4Raw with minimal modification. For each repetition, the reference images were formed by 2D inverse Fourier transform of the k-space data and taking root sum of squares of the coil channels. The reference images were also stored in the H5 files as potential training targets of parallel imaging reconstruction.

It should be noted that, similar to other MRI k-space datasets^30,31,33^, in order to preserve the raw data characteristics, the images were not further defaced. However, unlike 3D isotropic MRI data^25–27,31^, our 2D multi-slice data have relatively thick slices (5 mm thickness + 1 mm gap) covering only the upper half of the head (FOV_z_ = 108 mm), which renders potential facial identification through 3D reconstruction highly improbable.

### Subset partition

The dataset was divided into three subsets: training, validation, and motion-corrupted. The motion-corrupted subset was first extracted by identifying intra-scan and inter-scan motion. First, all data were visually inspected for severe intra-scan motion and apparent inter-scan motion. Then, the remaining data were examined quantitatively for inter-scan motion again. 3D translational motion model was employed with the parameters estimated using the Python scikit-image package^36^. Inter-scan motion was further divided into inter-repetition motion (between different repetitions of the same contrast), and inter-contrast motion (between the multi-repetition averaged images of different contrasts). Inter-repetition motion was considered severe if the translation was more than 1.25 pixels in-plane or 0.2 slice-thicknesses through-plane beyond the global means. Inter-contrast motion was considered severe if the translation was more than 5 pixels in-plane or 1 slice-thickness through-plane beyond the global means. As a result, 26 subjects were placed in the motion-corrupted subset because at least one of their scans contained severe motion following the abovementioned criterion. Last, the remaining data were randomly split into a training subset of 128 subjects (1024 volumes) and a validation subset of 30 subjects (240 volumes).

Note that in the above process, we only estimated the motion without performing actual correction for several reasons. Firstly, as shown in Table 2, the motion in the training and validation data was minor. Secondly, not all studies require motion correction; for example, inter-repetition motion has little impact on reconstruction of a single repetition, and inter-contrast motion should not interfere with most reconstruction algorithms unless multi-contrast strategies are employed^24,37–39^. Lastly, the optimal motion correction approach can vary depending on the specific application, including the choice of motion models and interpolation methods, which the M4Raw users may readily implement based on their own needs.

**Table 2:** Inter-scan head motion estimation (mean ± std, in pixels).

Direction	Inter-repetition motion			Inter-contrast motion		
	T1w	T2w	FLAIR	T1w-T2w	T1w-FLAIR	FLAIR-T2w
Slice encoding (Superior-inferior)	−0.01 ± 0.03	0.00 ± 0.02	−0.02 ± 0.04	−0.08 ± 0.20	−0.09 ± 0.16	0.01 ± 0.09
Frequency encoding (Anterior-posterior)	−0.39 ± 0.26	−0.31 ± 0.18	−0.31 ± 0.17	−1.50 ± 0.66	−0.83 ± 0.40	−0.64 ± 0.30
Phase encoding (Left-right)	0.01 ± 0.27	−0.01 ± 0.17	0.04 ± 0.21	0.14 ± 0.77	0.08 ± 0.61	0.04 ± 0.35

## Data Records

The multi-channel k-space and single-repetition images from the 183 participants, including T1-weighted, T2-weighted, and FLAIR contrasts, have been made publicly available through the Zenodo repository^40^. The training, validation, and motion-corrupted subsets are separately compressed into three zip files, containing 1024, 240, and 200 H5 files, respectively. Among the 200 files in the motion-corrupted subset, 64 files are placed in the “inter-scan_motion” sub-directory and 136 files in the “intra-scan_motion” sub-directory.

All the H5 files are named in the format of “study-id_contrast_repetition-id.h5” (e.g., “2022061003_FLAIR01.h5”). In each file, the imaging parameters, multi-channel k-space, and the single-repetition images can be accessed via the dictionary keys of “ismrmrd_header”, “kspace”, and “reconstruction_rss”, respectively. The k-space dimensions are arranged in the order of slice, coil channel, frequency encoding, and phase encoding, following the convention of the fastMRI dataset^30^.

## Technical Validation

### M4Raw dataset quality assessment

The quality of the data in the training and validation subsets was evaluated with regard to the image SNR, head motion, and coil sensitivity quality.

**Fig. 2:**
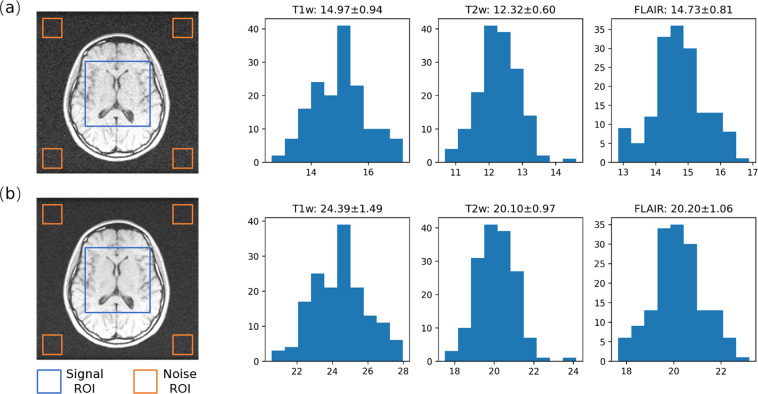
Signal-to-noise ratio (SNR) analysis of the training and validation data. (**a**) Representative image and SNR distribution of the single-repetition images. (**b**) Representative image and SNR distribution of multi-repetition averaged images. The signal and noise regions-of-interest (ROI) used for SNR calculation are indicated with blue and orange boxes, respectively.

The SNR of both the single-repetition images and the multi-repetition averaged images were measured by calculating the mean signal divided by the standard deviation of noise. As shown in Fig. 2, a signal region-of-interest (ROI) of size 100 × 100 was selected at the center of each image, while four noise ROIs of size 30 × 30 were selected at the corners. The resulting SNR distribution for each contrast is also plotted in Fig. 2. Overall, the SNRs of single-repetition images were 14.97 ± 0.94 for T1w, 12.32 ± 0.60 for T2w, and 14.73 ± 0.81 for FLAIR; the SNRs of multi-repetition averaged images were 24.39 ± 1.49 for T1w, 20.10 ± 0.97 for T2w, and 20.20 ± 1.06 for FLAIR. The ratios between the SNRs of single-repetition images and multi-repetition averaged images were 1.63 ± 0.02 for T1w, 1.63 ± 0.01 for T2w, and 1.37 ± 0.01 for FLAIR, which are close to their theoretical values, i.e., the square root of the number of repetitions.

The inter-scan head motion in the training and validation data was evaluated by 3D image translation estimation using the scikit-image package^36^. The results, presented in Table 2, indicated that the inter-repetition motion was minor, with standard deviation less than 0.3 pixels in-plane and 0.05 slice-thicknesses through-plane. The inter-contrast motion was slightly larger, yet still small, with standard deviation less than 0.8 pixels in-plane and 0.2 slice-thicknesses through-plane. All estimates had a near-zero mean, except for those in the frequency encoding direction. This was expected because the main field drift commonly observed in low-field systems with permanent magnets^3^ can cause slight shifting of the image in this direction over time.

**Fig. 3:**
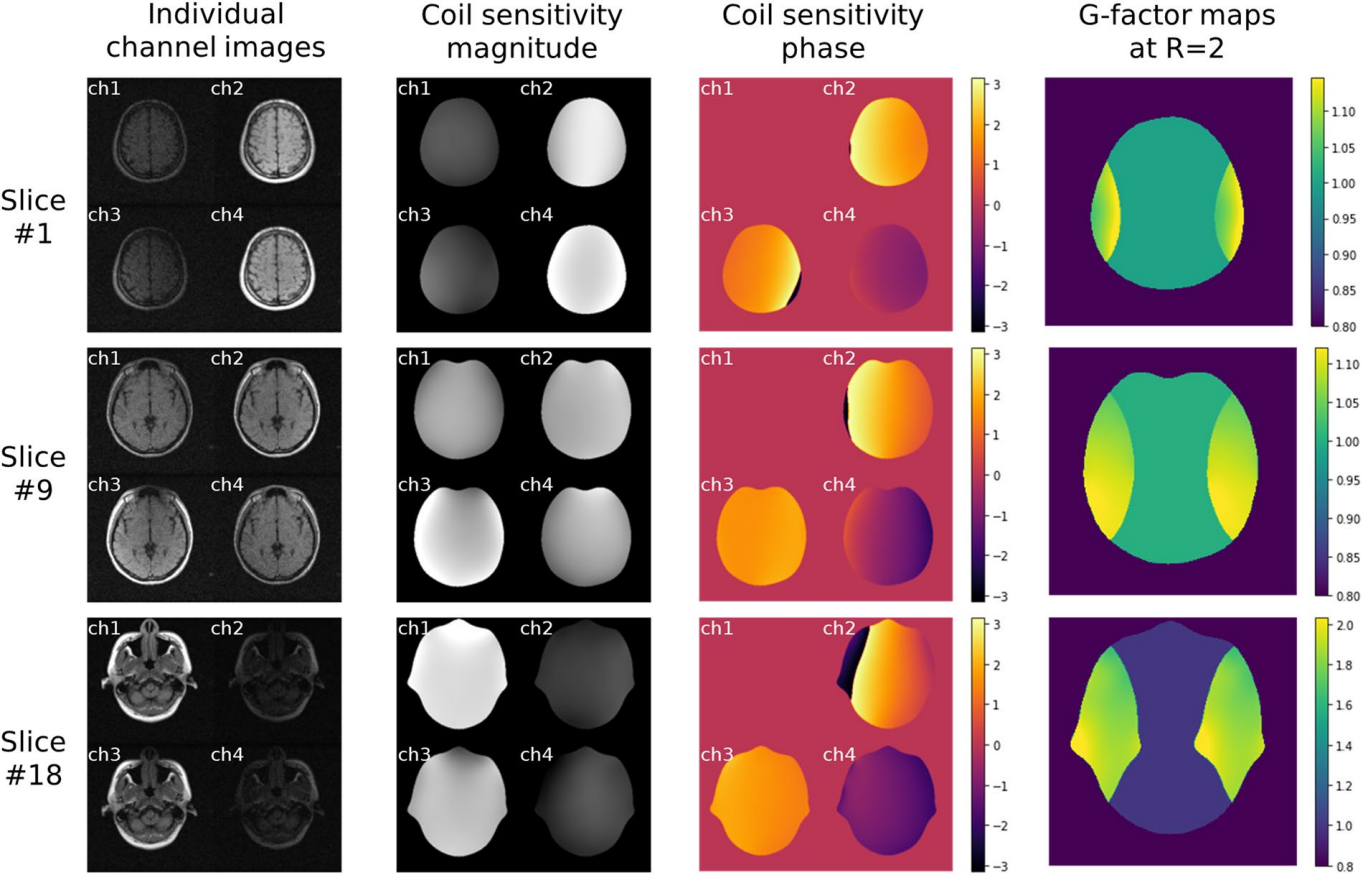
Representative images of individual coil channels, coil sensitivity maps, and the g-factor maps at acceleration factor (R) = 2.

**Fig. 4:**
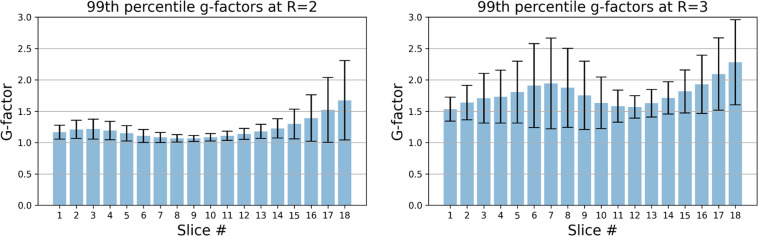
The 99th percentile g-factors on different slices as a measure of the coil sensitivity quality. The 99th percentile g-factors had a global mean ± std of 1.21 ± 0.29 at R = 2 and 1.78 ± 0.50 at R = 3, and the slice-wise mean values ranged from 1.07 to 1.67 at R = 2 and from 1.53 to 2.28 at R = 3. The high g-factors at R = 3 make it challenging for traditional parallel imaging methods to deliver useable images, yet data-driven methods may remain robust as illustrated in Fig. 5.

The coil sensitivity quality was quantitatively evaluated using the g-factors^41^, which are the pixel-wise noise amplification ratios in traditional SENSE reconstruction. The coil sensitivity maps were estimated using the ESPIRiT^42^ method by the Berkeley Advanced Reconstruction Toolbox (BART) toolbox (https://mrirecon.github.io/bart) and the g-factor maps were subsequently derived using the pygrappa package (https://github.com/mckib2/pygrappa). Figure 3 illustrates typical coil channel images, coil sensitivity maps, and g-factor maps at acceleration factor (R) = 2 for upper, middle, and lower slices. Figure 4 presents a statistical analysis of the 99th percentile g-factor values for different slices, which serves as a measure of maximum noise amplification. Overall, the 99th percentile g-factors had a global mean ± std of 1.21 ± 0.29 at R = 2 and 1.78 ± 0.50 at R = 3, while the slice-wise mean values ranged from 1.07 to 1.67 at R = 2 and from 1.53 to 2.28 at R = 3. The results demonstrated the feasibility of applying parallel imaging, but also indicated that traditional reconstruction methods^41,43^ might be seriously challenged at high acceleration factors.

### Demonstration of M4Raw dataset for parallel imaging reconstruction

**Fig. 5:**
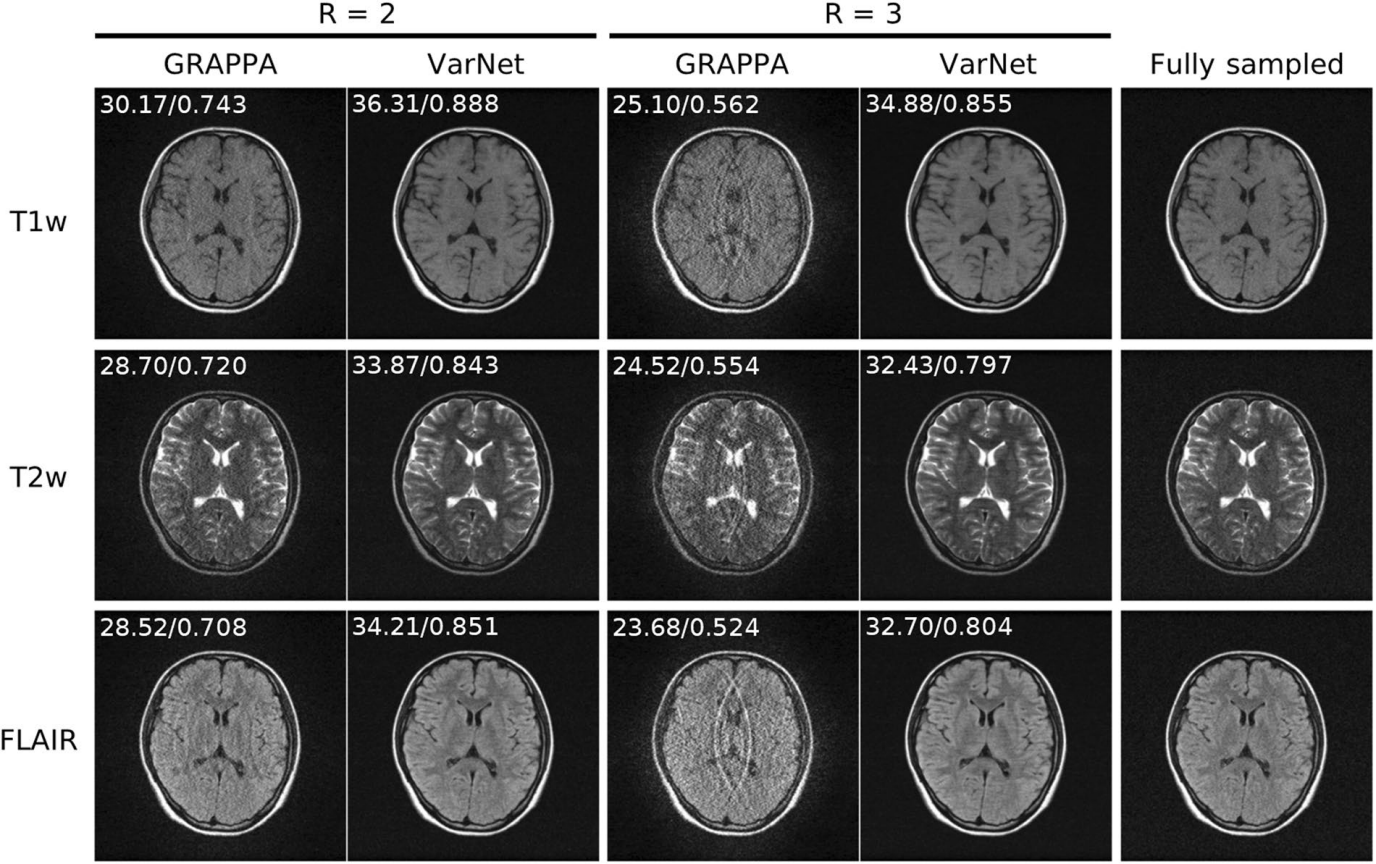
Results of the pilot M4Raw dataset demonstration for data-driven parallel imaging reconstruction. The mean PSNR and SSIM values were calculated for each contrast on the validation subset and labeled on top of the representative images. The trained VarNet model substantially outperformed the traditional GRAPPA method.

To demonstrate the utility of parallel imaging on the M4Raw dataset, an end-to-end variational network (VarNet) model^22^ was trained using the code from the fastMRI repository. Cartesian undersampling was retrospectively applied in the phase encoding direction at acceleration factors (R) = 2 and 3, while the central 256 × 30 k-space was left fully sampled for coil sensitivity calibration. The Adam optimizer was used with a learning rate of 1 × 10^−3^ for training over 50 epochs. The obtained model was evaluated on the validation subset and compared with the classical GRAPPA algorithm^43^ in terms of peak signal-to-noise ratio (PSNR) and structural similarity index (SSIM). The results, shown in Fig. 5, demonstrated that the VarNet method exhibited superior PSNR performance and produced high-quality images even at R = 3, whereas the noise amplification problem in GRAPPA was too severe to provide usable images.

### Demonstration of M4Raw dataset for denoising reconstruction

**Fig. 6:**
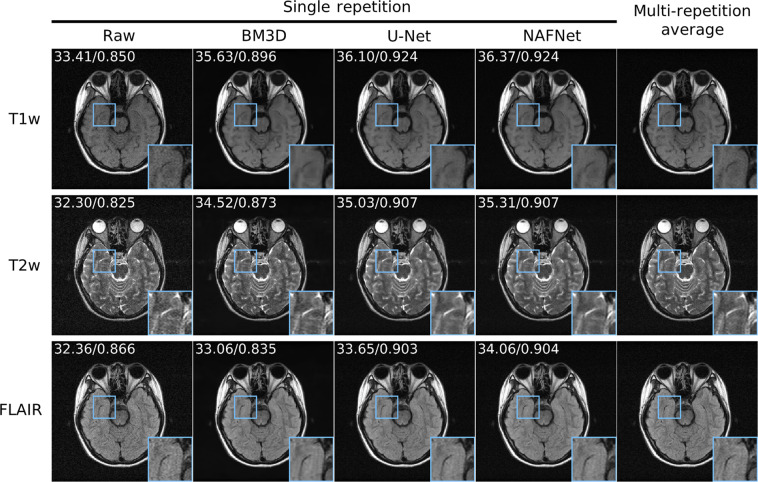
Results of the pilot M4Raw dataset demonstration for data-driven MRI denoising. The mean PSNR and SSIM values were calculated for each contrast on the validation subset and labeled on top of the representative images. Both data-driven methods achieved higher PSNR and SSIM values than the traditional BM3D method, offering visually improved images with less blurring.

For the demonstration of image denoising, a simple U-Net model^44^ and a state-of-the-art natural image restoration model NAFNet^45^ were trained on the M4Raw dataset. During training, the single repetition image was used as input, and the multi-repetition averaged image was used as the label. Both models were trained with Adam optimizer, and a learning rate of 1 × 10^−4^ was used for 50 training epochs before reduced to 1 × 10^−5^ for another 10 epochs. The trained models were then compared with the classical BM3D algorithm^46^, and the sigma parameter for BM3D was set to 0.025 according to the noise estimation. PSNR/SSIM values were computed for quantitative evaluation. As shown in Fig. 6, both data-driven methods outperformed the traditional BM3D method in terms of PSNR and SSIM values, and they offered visually better images with less blurring than the BM3D results.

## Usage Notes

The M4Raw dataset enables development of various data-driven methods for low-field MRI reconstruction. It can also serve as a benchmark dataset for comparing different methods specific to low-field MRI. The dataset encompasses characteristics of low-field MRI data, while also possessing similarities to existing high-field datasets^30–33^. As such, it can be used as a general test dataset for a wide range of MRI reconstruction algorithms, including those originally proposed for high-field MRI. Apart from parallel imaging and denoising, potential research applications for this dataset include super-resolution, motion correction, and image style transfer from low-field to high-field. Additionally, the dataset’s inclusion of multiple contrasts allows for joint multi-contrast image reconstruction to further advance image quality^24,37–39^.

For simplicity, researchers may opt to exclude the motion-corrupted subset from model training and evaluation. However, since it represents a common real-world MRI data imperfection, this subset can be valuable for developing motion correction techniques or motion-resistant reconstruction algorithms. It should be noted that not all data in this subset exhibit significant motion; if any scan from a subject was deemed motion-corrupted, all data related to that subject would be placed in this subset.

As can be observed from Fig. 3, the coil sensitivity exhibits large variations along the slice direction. Thus, another potential research application of this dataset is simultaneous multi-slice (SMS) reconstruction^47^. The k-space data can be multiplied by the CAIPIRINHA phase^48^ and summed along the slice direction to simulate SMS acquisition. SMS acceleration can effectively reduce the minimal TR and achieve higher SNR than in-plane acceleration. This technique is particularly suitable for low-field MRI without the SAR problem faced at high field^1^.

It should be noted that all M4Raw data were acquired using one MRI system, while other low-field systems may have different designs of magnets and coils^1–3^. Expanding the dataset to include data from a variety of low-field systems as well as paired data from high-field systems is an area of ongoing work. We welcome any collaborations to expand the dataset’s scope.

## Data Availability

To facilitate users of this dataset, we have released the following Github repository: https://github.com/mylyu/M4Raw. The repository contains Python examples for data reading and deep learning model training, and the trained model weights to reproduce the results in Figs. 2–6.
